# Lipocalin-2 and calprotectin as stool biomarkers for predicting necrotizing enterocolitis in premature neonates

**DOI:** 10.1038/s41390-021-01680-7

**Published:** 2021-08-31

**Authors:** Marie-Pier Thibault, Éric Tremblay, Chantal Horth, Aube Fournier-Morin, David Grynspan, Corentin Babakissa, Emile Levy, Emanuela Ferretti, Valérie Bertelle, Jean-François Beaulieu

**Affiliations:** 1 Laboratory of Intestinal Physiopathology, Faculty of Medicine and Health Sciences, Université de Sherbrooke, Sherbrooke, QC Canada; 2 Centre de Recherche du Centre Hospitalier Universitaire de Sherbrooke, Sherbrooke, QC Canada; 3 Division of Neonatology, Department of Pediatrics, Children’s Hospital of Eastern Ontario (CHEO) and CHEO Research Institute, Ottawa, ON Canada; 4 Department of Pathology and Laboratory Medicine, Faculty of Medicine, University of British Colombia, Vancouver, BC Canada; 5 Department of Pediatrics, Faculty of Medicine and Health Sciences, Université de Sherbrooke, Sherbrooke, QC Canada; 6 Research Center, Centre Hospitalier Universitaire Ste-Justine, Université de Montréal, Montréal, QC Canada; 7 Division of Neonatology, Department of Pediatrics, Faculty of Medicine and Health Sciences, Université de Sherbrooke, Sherbrooke, QC Canada

## Abstract

**Background:**

Necrotizing enterocolitis (NEC) is a major challenge for premature infants in neonatal intensive care units and efforts toward the search for indicators that could be used to predict the development of the disease have given limited results until now.

**Methods:**

In this study, stools from 132 very low birth weight infants were collected daily in the context of a multi-center prospective study aimed at investigating the potential of fecal biomarkers for NEC prediction. Eight infants (~6%) received a stage 3 NEC diagnosis. Their stools collected up to 10 days before diagnosis were included and matched with 14 non-NEC controls and tested by ELISA for the quantitation of eight biomarkers.

**Results:**

Biomarkers were evaluated in all available stool samples leading to the identification of lipocalin-2 and calprotectin as the two most reliable predicting markers over the 10-day period prior to NEC development. Pooling the data for each infant confirmed the significance of lipocalin-2 and calprotectin, individually and in combination 1 week in advance of the NEC clinical diagnosis.

**Conclusions:**

The lipocalin-2 and calprotectin tandem represents a significant biomarker signature for predicting NEC development. Although not yet fulfilling the “perfect biomarker” criteria, it represents a first step toward it.

**Impact:**

- Stool biomarkers can be used to predict NEC development in very low birth weight infants more than a week before the diagnosis.
- LCN2 was identified as a new robust biomarker for predicting NEC development, which used in conjunction with CALPRO, allows the identification of more than half of the cases that will develop NEC in very low birth weight infants.
- Combining more stool markers with the LCN2/CALPRO tandem such as PGE2 can further improve the algorithm for the prediction of NEC development.

## Introduction

One of the most life-threatening conditions for premature infants in neonatal intensive care units (NICU) is necrotizing enterocolitis (NEC), a gastrointestinal disease associated with severe intestinal inflammation and necrosis, which occurs in 5–16% of very low birth weight (VLBW) infants^1–4^ with a mortality rate of 20–50%^5–7^ and various long-term clinical sequels for the survivors.^7–10^ There are numerous factors that could be involved in the etiology and pathogenesis of NEC, but the most consistently reported ones are functional and immune intestinal immaturity and dysbiosis, formula feeding and low birth weight.^4,11–16^

NEC diagnosis is a challenge because it still relies on clinical and radiologic characteristics defined four decades ago,^17^ which do not allow it to be accurately distinguished from other intestinal conditions of distinct etiology.^13^ The efforts that have been made over the last five years toward the characterization of specific biomarkers for improving NEC diagnostic capabilities have led to the identification of a few potential candidates for distinguishing NEC from other similar pathological conditions.^18–21^ Considering the consensus among experts that the ideal biomarker should be at least non-invasive and identify VLBW infants most at risk of developing NEC prior to clinical presentation, further research is needed for its identification.^18,20–22^ Indeed, early detection of NEC would provide a period allowing targeted interventions such as antibiotic treatment or even administration of human donor milk.^11,13,18,23^ However, most studies have performed biomarker testing in samples from premature infants at the time of NEC diagnosis.^18,20,22^ For instance, fecal calprotectin (CALPRO), which is a member of the S-100 family used as an indicator of intestinal inflammation in the adult, has been investigated in several studies as a biomarker for NEC. In a recent meta-analysis including over 600 neonates, 10 of the 13 studies reported elevated levels of CALPRO among infants diagnosed with NEC, but its significance as an early screening marker remains unknown.^24^ The few exceptions include urinary intestinal-fatty acid binding protein that was shown to predict NEC one day before clinical manifestations.^25^ A combination of urinary biomarkers was also shown to predict disease severity in the first 6 h of NEC suspicion.^26^ Unfortunately, urinary samples can be difficult to collect in a standard NICU context and are considered an invasive method in the context of such a vulnerable and immunocompromised patient population, while blood sampling in preterm neonates is not recommended.^27^

In the present study, stools from VLBW infants were collected in the context of a multi-center prospective study aimed at investigating the predictive potential of fecal biomarkers for NEC.

## Methods

### Participants and samples

For this study, all premature infants admitted to the NICUs of the Centre Hospitalier Universitaire de Sherbrooke (Sherbrooke, QC) and the Ottawa Hospital General Campus (Ottawa, ON) with a birth weight less than 1500 g and born younger than 30 weeks of gestational age and for which a signed written informed consent was obtained from a parent or guardian were enrolled. Exclusion criteria were infants with birth age greater than 30 weeks, and infants with a documented congenital malformation of the intestinal tract (i.e. omphalocele, gastroschisis, malrotation, intestinal atresia, or congenital infracted segments). Preanalytical and postanalytical protocols were reviewed and approved by the institutional Ethics Review Board on Human Health Research of both hospitals.

Stools were collected daily by registered nurses at the bedside of 132 recruited infants all along their stay in the NICU and stored at −20 °C for 1–4 weeks until moved to −80 °C for long-term storage. Samples were assigned a numeric code for deidentification purposes and information about date of birth, mode of delivery, sex, gestational age at birth, birth weight, date of collection of diapers, and diagnosis. Overall, eight infants of the cohort received a stage 3 NEC diagnosis based on the clinical and radiologic criteria of Bell^17^ while 54 participants with no specific gastrointestinal conditions during their stay were included in the non-NEC control group. Stool samples corresponding to the 10 days prior to NEC diagnosis were identified as −10 to −1 and the sample on the day of diagnosis was identified as day 0 for each of the seven NEC series as one of the series collected over the 10-day period preceding diagnosis was too sparse and was therefore not included in the study. For each of the remaining NEC series, two non-NEC series of stool samples corresponding to 11 consecutive days were matched based on sex, gestational age, type of delivery, and birth weight. The clinical characteristics of the pairings are summarized in Table 1.

**Table 1:** Basic demographic and main clinical features of the NEC patients and non-NEC patients.

Pairing^a^		No.^b^	Gestational^c^		Birth weight (in grams)	Type of delivery	Feeding practice^d^	Antibiotics^e^	PDA^f^	Steroids^g^	
			Age	Sex						Prenatal	Postnatal
1	NEC	40	25 + 5	M	850	C/S	BM	Y	N	Y	N
	Non-NEC	33	26 + 0	M	850	C/S	BM	Y	N	Y	N
	Non-NEC	54	25 + 2	M	830	C/S	BM	N	N	Y	Y
2	NEC	43	26 + 4	F	930	C/S	F^h^	Y	Y	N	N
	Non-NEC	34	25 + 6	F	770	C/S	F^h^	Y	N	Y	N
	Non-NEC	128	26 + 0	F	860	C/S	BM/F^h^	Y	N	Y	N
3	NEC	55	26 + 3	F	960	VD	BM/F^h^	Y	N	Y	Y
	Non-NEC	53	26 + 5	F	1130	VD	F^h^	Y	Y	Y	N
	Non-NEC	54	25 + 1	F	730	VD	BM/F^h^	Y	Y	Y	N
4	NEC	57	26 + 4	M	1040	VD	F^h^	Y	Y	Y	N
	Non-NEC	31	26 + 1	M	920	VD	n/a^h^	N	Y	Y	N
	Non-NEC	125	26 + 5	M	1020	VD	BM/F^h^	N	Y	N	N
5	NEC	90	25 + 4	M	850	VD	BM/F^h^	Y	Y	Y	N
	Non-NEC	13	25 + 5	M	970	VD	BM/F^h^	Y	Y	Y	N
	Non-NEC	41	24 + 6	M	780	VD	n/a^h^	Y	Y	Y	N
6	NEC	96	25 + 4	M	890	VD	BM^h^	Y	Y	Y	N
	Non-NEC	42	26 + 4	M	921	VD	F^h^	Y	Y	Y	N
	Non-NEC	94	25 + 3	M	735	VD	BM^h^	Y	Y	Y	Y
7	NEC	118	24 + 4	M	660	VD	F^h^	Y	N	Y	Y
	Non-NEC	44	23 + 6	M	680	VD	BM/F^h^	Y	N	Y	Y
	Non-NEC	122	24 + 1	M	690	VD	BM/F^h^	Y	Y	Y	Y
	NEC	Av:	25 + 6		882.9						
	Non-NEC	Av:	25 + 4		849.0						

*C/S* cesarian, *VD* vaginal delivery, *Av* average.

^a^Two non-NEC control set of samples matched with each NEC sample set.

^b^Patient consecutive ID number.

^c^Age as weeks + days and sex as female (F) vs male (M).

^d^*BM* breast milk, *F* formula, *n/a* data nonavailable.

^e^Y: antibiotics at any time before or during stool collection; N: no antibiotic treatment.

^f^Y: indomethacin or ibuprofen treatment for patent ductus arteriosus (PDA); N: no treatment.

^g^Y: administration of steroids before or after birth; N: no steroid administration.

^h^Indicate that probiotics were included.

### Sample preparation and testing

Samples were prepared for ELISA testing using the Faecal Sample Preparation Kit (Buhlmann Smart-Prep; ALPCO Immunoassays, Salem, MA) by introducing 50–100 mg of the stools to 2.0–4.0 ml of extraction buffer in the sample chamber (~25 mg/ml), vortexed 1 min, and centrifugated at 3000 × *g* for 5 min. Supernatants were recovered, aliquoted, and frozen at −80° until later use for analysis.

Calprotectin (CALPRO) ELISA (ALPCO cat # 30-CALHU-E01), prostaglandin E2 (PGE2) ELISA (ALPCO cat # 74-PG2HU-E05), hemaglobin/haptoglobin complex (HAPTO-HEMO) ELISA (ALPCO cat# 30-HAPHU-E01), lysozyme (LYZ) ELISA (ALPCO Cat # 30-LYSHU-E01), osteoprotegerin (OPGN) ELISA (Biomedica Medizinprodukte, Cat# BI-20403, Vienna, Austria), and lipocalin-2 (LCN2/NGAL) ELISA (Epitope Diagnostics Inc., Cat# KRT-853, San Diego, CA) were all provided at no cost by ALPCO Immunoassay. Haptoglobin (HAPTO) DuoSet ELISA was from R&D Systems (Cat # DY8465-05, R&D Systems, Minneapolis, MN) and the Intestinal Alkaline Phosphatase (ALP) Activity Assay Kit was from Abcam (Cat # ab83371). All immunoassays were performed according to the manufacturer’s specific instructions.

For intestinal ALP protein analysis, proteins in samples were quantified by Pierce’s BCA Protein Assay (Thermo Fisher Scientific, Ottawa, ON) and prepared as 1 µg of stool protein/100 µl in Laemmli buffer under reducing conditions, separated on 12% sodium dodecyl sulfate polyacrylamide gels and transferred onto nitrocellulose for the detection of intestinal alkaline phosphatase using anti-human intestinal ALP (Abcam, Cat# ab186422) according to the procedures described previously^28^ and detected and quantified with a Li-Cor Odyssey Imaging System (Mandel Scientific, Guelph, ON).

### Data presentation and statistical analyses

Biomarkers were evaluated by ELISA in all available samples collected over the 11-day period for the 7 NEC and 14 non-NEC infants. Differences between NEC and non-NEC were evaluated with the Wilcoxon matched pairs signed-rank test. Change over the 10-day period preceding NEC diagnosis was evaluated by the simple linear regression goodness of fit test. Receiver operating characteristic (ROC) curves were also calculated for evaluating area under the ROC curve and cut-off values. NEC samples were then pooled for each infant according to three periods relative to the day of diagnosis: group A, 10 to 7 days before; group B, 6 to 3 days before, and group C, 2 days before to the day of the diagnosis and compared to non-NEC using the Wilcoxon matched pairs signed-rank test. Calculations and statistics were performed with Graph Pad Prism 9.0 (Graph Pad Software, San Diego, CA). *P* value <0.05 was considered significant unless otherwise specified.

## Results

**Fig. 1:**
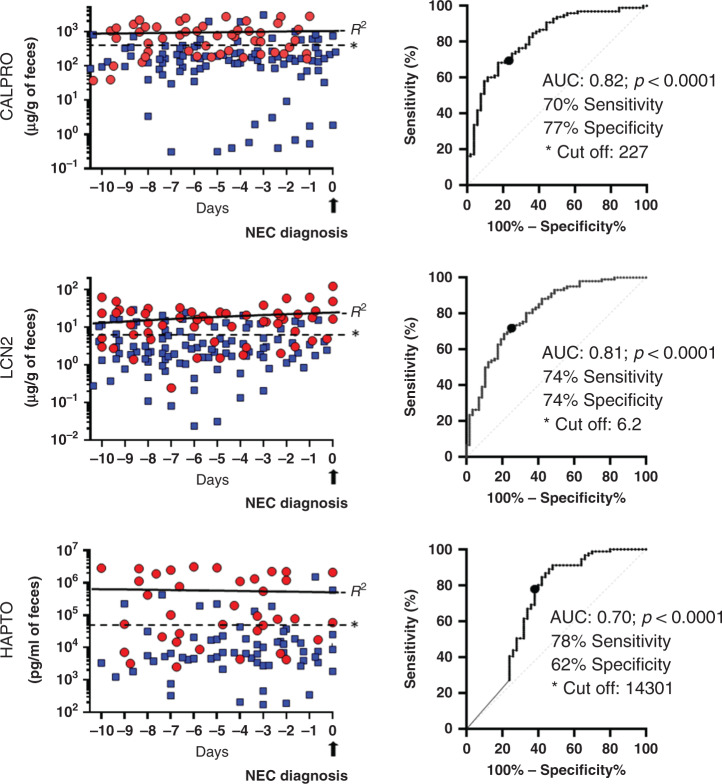
Detection of CALPRO, LCN2, and HAPTO in the stools of NEC and non-NEC premature infants by ELISA. Stool samples analyzed were those collected over a 10-day period preceding NEC diagnosis (round symbols) and in matched non-NEC infants (square symbols). Simple linear regression for the levels of biomarkers in the stools were calculated for both NEC and non-NEC with *R*^2^ values varying between 0.02 and 0.0003 while the slopes were considered nonsignificant for all. For simplicity, only the regression line (black line, *R*^2^) for NEC samples are shown (left panels). The biomarker data were then subjected to ROC curve analyses (right panels) showing sensitivity and specificity values and cut-off evaluation (dotted lines *, left panels).

Samples harvested from the seven NEC VLBW infants over the 11-day window corresponding to the day of diagnosis and the 10 days preceding it, as well as those of the matched non-NEC premature infants, were all tested by ELISA for the detection of potential biomarkers for intestinal disorders that could predict NEC development. As shown in Fig. 1 for CALPRO, LCN2, and HAPTO, variations in the levels of expression were seen for all three markers for both NEC and non-NEC samples but an apparent tendency for overexpression was noted for samples obtained from NEC infants (Fig. 1, left panels) and this tendency appeared to be relatively constant throughout the 10-day period preceding the diagnosis as confirmed by linear regression analysis (Fig. 1, black line in left panels). To further document this result, ROC curves were calculated for each marker to determine the area under the curve (AUC) and estimate a cut-off value. As shown on Fig. 1 (right panels), AUC for CALPRO and LCN2 were above 0.8 with sensitivities and specificities at 70% or higher confirming that these two biomarkers are consistently increased in stool samples of infants with NEC in the 10-day period preceding diagnosis. A statistically significant increase in HAPTO was also noted while the AUC for the other tested markers were below 0.7 and therefore not further considered for these analyses.

**Fig. 2:**
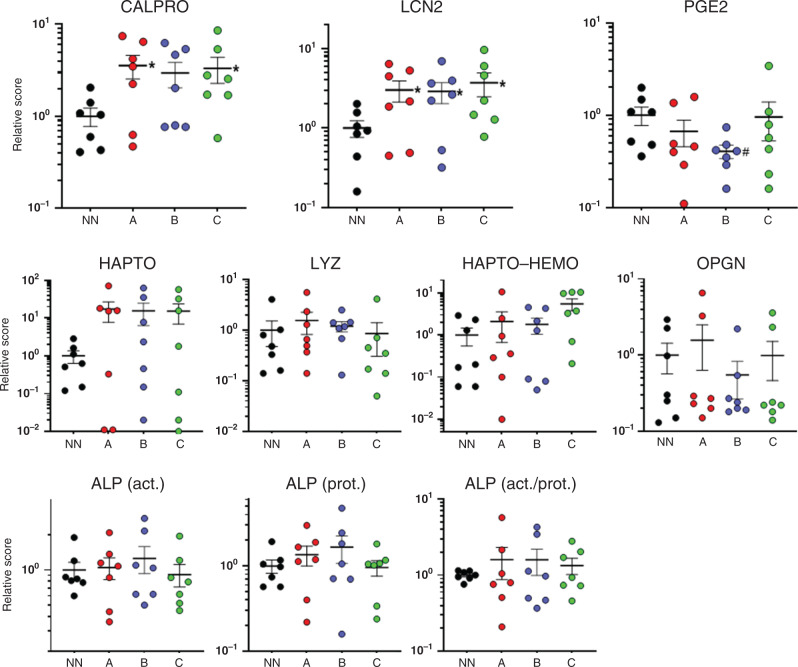
Relative levels of biomarkers in the stools of NEC infants for distinct periods preceding diagnosis. For each NEC infant, the stool samples were pooled for three periods relative to the diagnosis: group A, −10 to −7 days; group B, −6 to −3 days; and group C, −2 to 0 days while non-NEC samples (NN) were pooled for the 10-day period. The means ± SEM are illustrated. *P < 0.05 (^#^*P* < 0.07) using the Wilcoxon matched pairs signed-rank test by comparison with NN.

As expected from the ROC curves, a majority of the NEC samples were found to be above the cut-off value established for each marker, and at all stages (Fig. 1, dotted line in left panels) suggesting that these biomarkers can discriminate premature infants that will develop NEC more than a week in advance. To further explore this possibility, stool samples from the seven NEC infants were pooled according to the time obtained before diagnosis: group A: −10 to −7; group B: −6 to −3, and group C: −2 to 0 (Fig. 2). Data from each of the two controls for the seven paired sets of samples were also pooled for statistical purposes. It is noteworthy that the detection of all tested biomarkers was found to be constant toward the 10-day period as evaluated by linear regression analyses. Then, considering the differences in the range of expression for each marker varying from several orders of magnitude, individual data were expressed according to a relative score where for each of the tested markers, the average of controls was calculated as 1.0 in order to allow comparisons between markers and evaluate the potential of multiplex combinations as addressed below. As shown in Fig. 2, the expression of each marker for the seven NEC infants relative to controls is presented. CALPRO and LCN2 are significantly increased in stool samples of NEC infants up to 1 week before the diagnosis. No other markers showed statistical significance. Individually, three out of the seven NEC patients would have been predicted by the higher level of expression of CALPRO, LCN2 or HAPTO. Expression levels of LYZ, HAPTO-HEMO, OPGN, or ALP were not predictive individually but a tendency to reduction (*P* < 0.07) for PGE2 was noted in group B (Fig. 2).

**Fig. 3:**
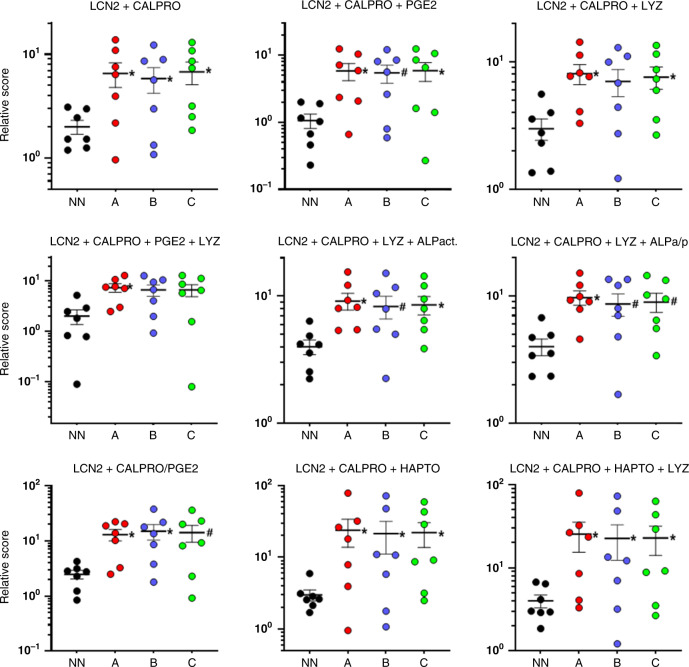
Relative levels of various combinations of biomarkers in the stools of NEC infants for distinct periods preceding diagnosis. For each NEC infant, the stool samples were pooled for three periods relative to the diagnosis: group A, −10 to −7 days; group B, −6 to −3 days; and group C, −2 to 0 days while non-NEC samples (NN) were pooled for the 10-day period and the data for combining the biomarkers were processed as indicated. The means ± SEM are illustrated. *P < 0.05 (^#^*P* < 0.07) using the Wilcoxon matched pairs signed-rank test by comparison with NN.

Based on recent evidence that multiplexing can be advantageous for improving diagnosis, various combinations of markers were tested. The combination of the two best individual markers, CALPRO and LCN2, significantly improved the predictive value of the test by identifying four of the seven NEC patients for the three tested windows (Fig. 3). Further combinations to these two markers with one or two other markers including PGE2, LYZ, ALP, and HAPTO did not lead to a significant improvement since at least three of the NEC samples could not be distinguished from the controls. One exception was the combination of (CALPRO + LCN2)/PGE2 that allowed the prediction of five of the seven infants that developed NEC a minimum of 7 days in advance (Fig. 3).

## Discussion

Considering that NEC may have important short-term effects on VLBW infants as well as several long-term consequences for survivors, the need for accurately identifying the premature newborns that are most susceptible to developing the disease is more and more recognized as an important step in clinical management to facilitate preventive measures.^29^ Indeed, in addition to broad-spectrum antibiotic treatments, there are potential beneficial therapies that can be used to prevent NEC focussing on the repression of the inflammatory signaling pathways as well as the administration of probiotics, human milk, and factors that can accelerate intestinal maturation.^11,12,18,20,23,30^ For instance, epidermal growth factor (EGF) which appears to be a potential therapeutic agent for preventing NEC^31–33^ has been shown to exert anti-inflammatory, anti-oxidative, and pro-maturation effects on the immature human intestinal mucosa.^34–36^ Predicting which VLBW infants will develop NEC is therefore crucial for accurately testing these new therapies, as well as to avoid over-treating newborns that will not develop NEC since some of these therapies, such as antibiotic treatment for instance, are not without consequence for the newborns.^13^

Up to now, most studies investigated NEC-related biomarkers in blood, urine, or stool samples obtained at the time of NEC symptoms, thus leading to the identification of biomarkers that may improve the diagnosis although of limited predictive value for NEC development. As mentioned above, a few exceptions including urinary biomarkers were shown to be predictive 6–24 h before clinical symptoms in some cases but to our knowledge, no systematic prospective collection of samples obtained using non-invasive methods has been performed in the context of searching for NEC predictive biomarkers. In the present study, out of the 132 premature infants admitted into the NICU with a birth weight less than 1500 g and born younger that 30 weeks recruited for our study, 8 received a confirmed diagnosis of NEC, which overall represents ~ 6% of VLBW infants in agreement with previous reports.^1–4^ Considering that early NEC detection is one of the prime characteristics of the ideal biomarker,^21^ we included stool samples obtained up to 10 days before the diagnosis of NEC for the purpose of the biomarker search.

**Fig. 4:**
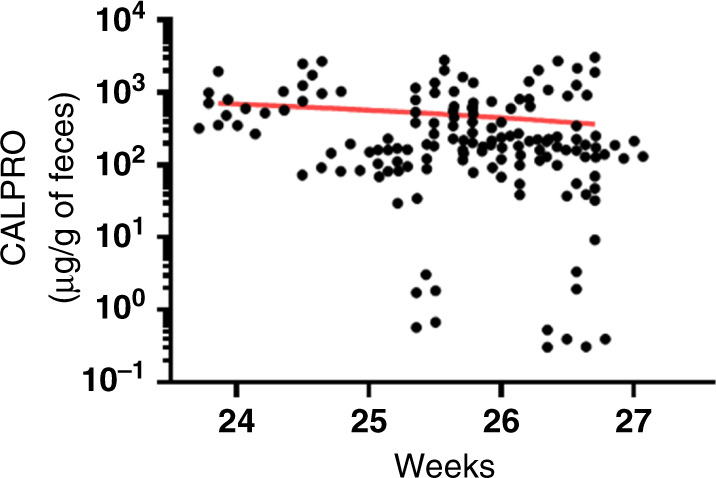
Detection of CALPRO in the stools of NEC and non-NEC infants according to their gestation ages. Stool samples analyzed were those collected in the period preceding NEC diagnosis and in matched non-NEC infants according to their gestational age. Simple linear regression was calculated with *R*^2^ squared = 0.023 and a slope considered nonsignificant.

Based on its wide use as a biomarker for inflammatory bowel disease, CALPRO has been one of the most investigated experimental NEC diagnosis markers.^24^ As reviewed recently,^19,20^ CALPRO usefulness for predicting NEC was questioned by the fact that its levels in the feces vary with gestational age, being higher in preterm than term neonates,^37^ which once considered may nevertheless improve the diagnosis of NEC.^38,39^ Stool samples from all NEC and their matched non-NEC controls tested herein were from a relatively narrow window, between 24 and 27 weeks and near or below 1000 g at birth. Analysis of the samples showed no overall correlation between gestational ages and fecal CALPRO concentrations (Fig. 4; *R*^2^ = 0.02316, NS). As expected from previous studies, higher levels of CALPRO were noted in the stools of premature infants near NEC diagnosis but more interestingly for this study, CALPRO levels were found to be higher in the stools of NEC infants 7–10 days before diagnosis, which would have allowed the prediction of NEC in half of the premature infants that consequently developed the disease. In a recent study that included infants at high-risk for NEC, CALPRO levels in the stools of the ten infants who developed NEC as well as the controls were found to be highly variable leading the authors to conclude that these variations preclude the use of CALPRO concentration for the early detection of NEC in high-risk infants.^40^ We speculate that three major differences between this study and our own are likely to be responsible for the different findings: (1) the NEC infants included in our study were at a lower gestational age and birth weight (25 weeks + 6 days and 883 g herein vs 27.5 weeks and 1010 g), (2) our control cohort was matched for gestational age, sex, and birth weight compared to an heterogeneous control groups including infants up to 32 weeks and infants born from mothers who had received indomethacin^40^, and lastly, (3) in our study, the collection of stool samples was done daily instead of twice a week as in the van Zoonen study^40^ allowing us to decrease variability.

The second biomarker investigated was LCN2, also known as neutrophil gelatinase-associated lipocalin, an anti-microbial molecule upregulated in intestinal cells under proinflammatory conditions.^41^ LCN2 was first demonstrated to be a potential biomarker for monitoring inflammatory bowel disease in the adult^42,43^ and was recently reported by our group to be a key marker associated with an ileal sample of NEC infants.^44^ Interestingly, the release of LCN2 in the stool of preterm infants was relatively comparable to that of CALPRO, being upregulated well before NEC diagnosis thus representing a potential new robust marker for this condition.

Prostaglandin E2 was the only other marker for which a modulation, although slight, was noted in the 6-3-day period preceding NEC diagnosis. It is noteworthy that none of the NEC infants received COX inhibitors during this period. Reduction of PGE2 under intestinal proinflammatory conditions may appear surprising at first sight considering that the COX pathway has been reported to be activated during chronic intestinal inflammation,^45,46^ but several lines of evidence indicate that PGE2 may be protective for the intestinal mucosa^47,48^ and that in fact it is the low levels of PGE2 that may predispose the patient to relapsing intestinal inflammation^49^ consistent with our observation of a reduction in the release of PGE2 before the appearance of NEC symptoms.

No significant variation was noted for other tested markers, including (i) LYZ, which as other Paneth cell products, has been reported to be increased in ileal samples of premature infants with NEC at the transcript level^44,50^ but not at the protein level;^50,51^ (ii) Haptoglobin detected as HAPTO or HAPTO-HEMO, a marker for small bowel lesions^52^ which has been found to prevent NEC injury in a red-blood cell transfusion murine model,^53^ and (iii) OPGN, a marker for intestinal inflammation in children.^54,55^ Furthermore, based on a recent study showing an association of intestinal ALP with NEC in premature infants where high amounts of ALP protein and low ALP enzyme activity were observed at the time of diagnosis,^56^ we investigated the same parameters but noted no significant change, suggesting that intestinal ALP is not a reliable predictive marker for NEC. However, it was reported by Heath et al.^56^ that a minimum of 12 patients diagnosed with NEC were needed to demonstrate significance.

Next, various combinations of biomarkers were assessed. First, the CALPRO–LCN2 combination improved the significance between controls and NEC samples for all three stages. It is interesting to note that the fecal CALPRO test is implemented in most biochemical clinical units for monitoring inflammatory bowel disease while LCN2 is mostly tested for kidney-related diseases^57^ although a number of commercial assays are available for its evaluation in stools. Inclusion of other tested markers had no additional effect considering that three of the NEC samples remained in the same range as the controls excepted for the ratio CALPRO + LCN2/PGE2 which would have led to the prediction of the occurrence of NEC in five of the seven patients a week in advance.

Although considering the relatively low number of NEC patients included in this proof-of-concept investigation, which is the main limitation of this study, these results warrant further investigation with a larger cohort of premature patients. One of the major strengths of the findings is the practical and non-invasive predictive nature of these identified biomarkers, which would allow weekly or bi-weekly stool sampling test in the VLBW population at risk for NEC. Identifying a large proportion of the most susceptible individuals at risk for developing NEC would facilitate different steps toward a true primary preventive intervention. This should be seen as a significant advantage in undertaking a larger multi-center trial. The fact that such a test may only identify 50–60% of the premature infants that will develop NEC (e.g. 4–5 out of 7, 8 if we include the potential constipation problematic in this population) can be seen as a weakness in the context of the “ideal biomarker” definition.^19,21^ However, considering the human and economic cost of each NEC case, one may consider that the benefit of identifying only a portion of the VLBW in the NICU and preventing its progression would counteract the cost of implementing such a test for the health system. However, this research was performed in the context of a biomarker discovery phase. Considering all other potential biomarkers and clinical features reported in the literature for NEC diagnosis,^18,20–22,44^ one would expect that further investigations using such prospective sample collection in NICU may lead to the discovery of additional predictive biomarkers and/or features that could complement our findings.

In conclusion, our study has led to the identification of LCN2 as a new robust biomarker for predicting NEC development, which used in conjunction with CALPRO would have allowed the identification of VLNW infants more than a week before the diagnosis in four out of the eight cases that developed NEC, and in five out of the eight cases if PGE2 was also included in the algorithm. While it would seem premature to recommend changes to the NICU practice based on these results, we hope that this practical and feasible approach of using multiple biomarkers will spark additional research to confirm these observations on larger cohorts of premature newborn patients. This will help to establish a trend for different gestational ages and validating the potential to implement a predictive test that would improve critical clinical observations and allow time for preventive interventions against the development of NEC.
